# Efficient Biexciton Preparation in a Quantum Dot—Metal Nanoparticle System Using On-Off Pulses

**DOI:** 10.3390/nano11071859

**Published:** 2021-07-19

**Authors:** Athanasios Smponias, Dionisis Stefanatos, Emmanuel Paspalakis

**Affiliations:** Materials Science Department, School of Natural Sciences, University of Patras, 26504 Patras, Greece; s_sbonias@hotmail.com (A.S.); paspalak@upatras.gr (E.P.)

**Keywords:** semiconductor quantum dots, biexciton, plasmonics, quantum control

## Abstract

We consider a hybrid nanostructure composed by semiconductor quantum dot coupled to a metallic nanoparticle and investigate the efficient creation of biexciton state in the quantum dot, when starting from the ground state and using linearly polarized laser pulses with on-off modulation. With numerical simulations of the coupled system density matrix equations, we show that a simple on-off-on pulse-sequence, previously derived for the case of an isolated quantum dot, can efficiently prepare the biexciton state even in the presence of the nanoparticle, for various interparticle distances and biexciton energy shifts. The pulse durations in the sequence are obtained from the solution of a transcendental equation.

## 1. Introduction

An active research topic within the field of quantum plasmonics [1,2] is the efficient population control of the exciton and biexciton states in semiconductor quantum dots (SQD) closely placed to metallic nanoparticles (MNP) [3,4,5,6,7,8,9,10,11,12,13,14,15,16]. For these hybrid nanostructures the population dynamics is rather different compared to the case of a single SQD, since the presence of the MNP amplifies the external electric field and induces interaction between SQD excitons and localized surface plasmons [17,18,19,20,21,22,23,24,25,26,27,28]. A great portion of studies in this area is devoted to population transfer between the ground and single exciton states of the SQD, using external fields and with the MNP present [3,4,5,6,7,8,9,10,12,13,14,16]. In many of these works it is reported that the MNP substantially modifies the period of Rabi oscillations between these states [3,4,5,6,7,8,9], which can even be destroyed for specific SQD-MNP distances [4,5,8,9]. High levels of population transfer to the exciton state can be achieved by applying carefully designed short [12,14,16] and ultra-short [13,14,16] pulses, while in another work the presence of MNP has been used to accomplish electromagnetically induced selective excitonic population transfer [10]. Additionally, optimal control has been exploited to improve population transfer between the lower energy levels of a Λ-type SQD coupled to a MNP [15], while a mixed scheme of pulsed and continuous wave fields has been recommended to efficiently prepare a single hole spin state in a SQD-MNP system [11].

The problem of population transfer from the ground to the biexciton state in a single SQD (without a MNP) has also become the center of immense research activity [29,30,31,32,33,34,35,36,37,38,39,40,41,42,43], with potential applications the efficient generation of single photons [44] and polarization-entangled photons [45,46,47,48,49], processes which can be exploited for various quantum information processing tasks [50]. A linearly polarized laser pulse is often used to implement the two-photon ground to biexciton transition. On the other hand and despite that the placement of a MNP next to the SQD has been suggested in order to enhance biexciton emission [51,52] and improve the production of polarization-entangled photons [53], there are not many works studying controlled biexciton dynamics in the SQD-MNP system. In Ref. [54] the two-photon Rabi oscillations in this system have been explored, while in our recent work [55] we showed how population transfer to the biecxiton state can be efficiently achieved by applying linearly polarized pulses with hyperbolic secant profile.

In this article, we also examine the problem of coherently preparing the biexciton state in a coupled SQD-MNP nanosystem, using on-off pulse-sequences that we have previously derived for an isolated SQD [43]. We use the three-level quantum cascade model for the SQD and the corresponding modified nonlinear density matrix equations, which also take into account the applied electromagnetic field as well as the interaction between MNP surface plasmons and SQD excitons [12,17,18,20,54,56]. The externally applied field has linear polarization and is at two-photon resonance with the ground to biexciton transition, while the pulses have a simple on-off-on modulation [43], where the durations of the on-off segments can be found by solving a transcendental equation. These control pulses are derived to quickly accomplish perfect biexciton state preparation in an *isolated* SQD and in the idealized case where relaxation processes are ignored. Here, we apply them in the coupled SQD-MNP nanostructure and show with numerical simulations of the complete system equations (including relaxation and interparticle interactions) that high-levels of fidelity can still be obtained for a wide range of biexciton energy shifts and a variety of SQD-MNP interparticle distances.

The structure of the paper is as follows. In Section 2 we provide the equations which describe the SQD-MNP system under the influence of the external electromagnetic field. In Section 3 we derive the on-off pulses used to populate the biexciton state, while in Section 4 we apply them in the equations of the coupled SQD-MNP system. In Section 5 we provide a summary of the results.

## 2. Coupled SQD-MNP System

**Figure 1:**
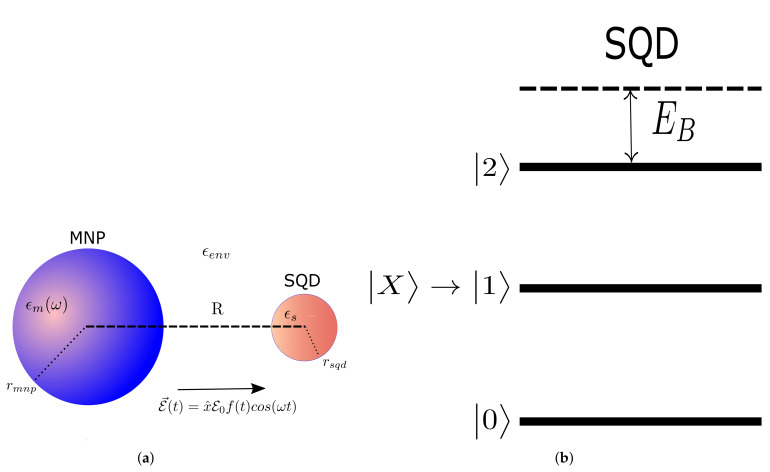
(**a**) Coupled semiconductor quantum dot (SQD)-metal nanoparticle (MNP) nanosystem. (**b**) Energy levels for the biexciton system.

In Figure 1 is shown the nanosystem that we study in this article. A spherical MNP with radius $r_{mnp}$ and a spherical SQD with radius $r_{sqd}$, are placed a distance $R>r_{mnp}$ from each other, in a dielectric environment with constant $\varepsilon_{env}$, taken to be real. A full account of the quantum dot is accomplished using a four-level system [32,33]. The corresponding energy levels include the ground state |0〉, the biexciton state |2〉 and, when we apply fields with linear polarization, the linearly polarized single-exciton states |X〉 and |Y〉. In this study we consider an *x*-polarized applied field which excites the path |0〉→|X〉→|2〉, while state |Y〉 is not involved. In order to comply with the naming of the other states, we denote state |X〉 with |1〉, thus the states participating in the ground to biexciton transfer are |0〉, |1〉, and |2〉. These states form the biexciton ladder three-level system, displayed in Figure 1. The applied field $\vec{E}\left(t\right)=\hat{x}E_{0}f\left(t\right)\cos\left(\omega t\right)$ excites in SQD the cascade transitions ground-exciton-biexciton, where $\hat{x},E_{0},f\left(t\right),\omega$ denote its polarization, amplitude, dimensionless envelope and center frequency, respectively. The SQD and MNP dielectric constants are denoted by $\varepsilon_{S},\varepsilon_{m}\left(\omega \right)$, respectively. Furthermore, the applied field stimulates surface plasmons on the MNP which come up with a powerful continuous spectral response, interact with SQD excitons [3,17,18,56] and thus couple the two nanoparticles, leading to Förster energy transferral [57].

Applying the dipole approximation, the biexciton cascade Hamiltonian becomes

(1) $$H_{B}\left(t\right)=E|1\rangle \langle 1|+\left(2E+E_{B}\right)\left|2\rangle \langle 2\right|-\mu E_{\mathrm{SQD}}\left(t\right)(|0\rangle \langle 1|+|1\rangle \langle 2|+H.c.),$$

where *E* denotes the exciton energy, with respect to zero ground state energy, $E_{B}$ the biexciton energy shift, μ the dipole moment for both ground-exciton and exciton-biexciton transitions, while $E_{\mathrm{SQD}}$ is the total electric field inside the SQD, originating from the external field as well as the induced field stemming from the MNP. In the quasistatic approximation the corresponding expression is [17,18,20,56]:

(2) $$E_{\mathrm{SQD}}\left(t\right)=\frac{\hbar }{\mu }\left[\frac{\Omega (t)}{2}e^{-i\omega t}+G\left[\rho_{10}\left(t\right)+\rho_{21}\left(t\right)\right]+H.c.\right]\;,$$

with $\rho_{ij}\left(t\right)$ being density matrix elements for the three-level system, the time-dependent Rabi frequency Ω(t) is [17,18,20]

(3) $$\Omega \left(t\right)=\Omega_{0}f\left(t\right)\;,\;\Omega_{0}=\frac{\mu E_{0}}{\hbar \varepsilon_{effS}}\left(1+\frac{s_{a}\gamma_{1}r_{mnp}^{3}}{R^{3}}\right)\;,$$

while parameter *G* is given by [18]

(4) $$G=\sum_{n=1}^{N}\frac{1}{4\pi \varepsilon_{env}}\frac{{\left(n+1\right)}^{2}\gamma_{n}r_{mnp}^{2n+1}\mu^{2}}{\hbar \varepsilon_{effS}^{2}R^{2n+4}}\;.$$

In the above expressions, $\varepsilon_{effS}=\frac{2\varepsilon_{env}+\varepsilon_{S}}{3\varepsilon_{env}}$, $\gamma_{n}=\frac{\varepsilon_{m}\left(\omega \right)-\varepsilon_{env}}{\varepsilon_{m}\left(\omega \right)+\left(n+1\right)\varepsilon_{env}/n}$ with *n* positive integer, while $s_{a}=2$ for an external field parallel to the interparticle SQD-MNP *x*-axis.

The two terms appearing in the Rabi frequency, Equation (3), are due to the external field and to the electric field of the MNP, which is induced from the external field. Furthermore, parameter *G* occures due to the excitons-plasmons interaction mentioned above [3,17,18,19]. To become more specific, the applied electric field induces a dipole on the SQD, inducing in turn a dipole on the MNP, wich subsequently affects the SQD via the self-interaction parameter *G* [17,20]. The expression of Equation (4) takes into account multipole effects and using f.e. N=20 terms produces more precise values of self-interaction *G* [18].

The time evolution of the density matrix for the biexciton cascade can be described by the following system

(5a) $$\begin{array}{ccc} {\dot{\rho }}_{00}\left(t\right) & = & \Gamma_{11}\rho_{11}\left(t\right)+i\frac{\mu E_{\mathrm{SQD}}\left(t\right)}{\hbar }\left[\rho_{10}\left(t\right)-\rho_{01}\left(t\right)\right]\;, \end{array}$$

(5b) $$\begin{array}{ccc} {\dot{\rho }}_{22}\left(t\right) & = & -\Gamma_{22}\rho_{22}\left(t\right)+i\frac{\mu E_{\mathrm{SQD}}\left(t\right)}{\hbar }\left[\rho_{12}\left(t\right)-\rho_{21}\left(t\right)\right]\;, \end{array}$$

(5c) $$\begin{array}{ccc} {\dot{\rho }}_{01}\left(t\right) & = & \left(i\frac{E}{\hbar }-\gamma_{01}\right)\rho_{01}\left(t\right)+i\frac{\mu E_{\mathrm{SQD}}\left(t\right)}{\hbar }\left[\rho_{11}\left(t\right)-\rho_{00}\left(t\right)\right]-i\frac{\mu E_{\mathrm{SQD}}\left(t\right)}{\hbar }\rho_{02}\left(t\right)\;, \end{array}$$

(5d) $$\begin{array}{ccc} {\dot{\rho }}_{02}\left(t\right) & = & \left(i\frac{2E+E_{B}}{\hbar }-\gamma_{02}\right)\rho_{02}\left(t\right)+i\frac{\mu E_{\mathrm{SQD}}\left(t\right)}{\hbar }\rho_{12}\left(t\right)-i\frac{\mu E_{\mathrm{SQD}}\left(t\right)}{\hbar }\rho_{01}\left(t\right)\;, \end{array}$$

(5e) $$\begin{array}{ccc} {\dot{\rho }}_{12}\left(t\right) & = & \left(i\frac{E+E_{B}}{\hbar }-\gamma_{12}\right)\rho_{12}\left(t\right)+i\frac{\mu E_{\mathrm{SQD}}\left(t\right)}{\hbar }\left[\rho_{22}\left(t\right)-\rho_{11}\left(t\right)\right]+i\frac{\mu E_{\mathrm{SQD}}\left(t\right)}{\hbar }\rho_{02}\left(t\right)\;, \end{array}$$

where $\sum_{i=1}^{3}\rho_{ii}\left(t\right)=1$ and $\rho_{nm}\left(t\right)=\rho_{mn}^{*}\left(t\right)$. In the above equations $\Gamma_{11}$, $\Gamma_{22}$ denote the decay rates for the exciton and biexciton energy levels, respectively, and $\gamma_{nm}$, with n≠m, the dephasing rates of the system. We proceed with a change of variables $\rho_{nn}\left(t\right)=\sigma_{nn}\left(t\right)$, $\rho_{01}\left(t\right)=\sigma_{01}\left(t\right)e^{i\omega t}$, $\rho_{02}\left(t\right)=\sigma_{02}\left(t\right)e^{2i\omega t}$, and $\rho_{12}\left(t\right)=\sigma_{12}\left(t\right)e^{i\omega t}$ and make the rotating wave approximation, in order to obtain the time evolution for the slowly varying envelopes of the density matrix elements:

(6a) $$\begin{array}{ccc} {\dot{\sigma }}_{00}\left(t\right) & = & \Gamma_{11}\sigma_{11}\left(t\right)+i\frac{\Omega^{*}\left(t\right)}{2}\sigma_{10}\left(t\right)-i\frac{\Omega (t)}{2}\sigma_{01}\left(t\right)\\ & + & iG^{*}\left[\sigma_{01}\left(t\right)+\sigma_{12}\left(t\right)\right]\sigma_{10}\left(t\right)-iG\left[\sigma_{10}\left(t\right)+\sigma_{21}\left(t\right)\right]\sigma_{01}\left(t\right)\;, \end{array}$$

(6b) $$\begin{array}{ccc} {\dot{\sigma }}_{22}\left(t\right) & = & -\Gamma_{22}\sigma_{22}\left(t\right)+i\frac{\Omega (t)}{2}\sigma_{12}\left(t\right)-i\frac{\Omega^{*}\left(t\right)}{2}\sigma_{21}\left(t\right)\\ & + & iG\left[\sigma_{10}\left(t\right)+\sigma_{21}\left(t\right)\right]\sigma_{12}\left(t\right)-iG^{*}\left[\sigma_{01}\left(t\right)+\sigma_{12}\left(t\right)\right]\sigma_{21}\left(t\right)\;, \end{array}$$

(6c) $$\begin{array}{ccc} {\dot{\sigma }}_{01}\left(t\right) & = & \left(i\frac{E}{\hbar }-i\omega -\gamma_{01}\right)\sigma_{01}\left(t\right)+i\frac{\Omega^{*}\left(t\right)}{2}\left[\sigma_{11}\left(t\right)-\sigma_{00}\left(t\right)\right]-i\frac{\Omega (t)}{2}\sigma_{02}\left(t\right)\\ & + & iG^{*}\left[\sigma_{01}\left(t\right)+\sigma_{12}\left(t\right)\right]\left[\sigma_{11}\left(t\right)-\sigma_{00}\left(t\right)\right]-iG\left[\sigma_{10}\left(t\right)+\sigma_{21}\left(t\right)\right]\sigma_{02}\left(t\right)\;, \end{array}$$

(6d) $$\begin{array}{ccc} {\dot{\sigma }}_{02}\left(t\right) & = & \left(i\frac{2E+E_{B}}{\hbar }-2i\omega -\gamma_{02}\right)\sigma_{02}\left(t\right)+i\frac{\Omega^{*}\left(t\right)}{2}\left[\sigma_{12}\left(t\right)-\sigma_{01}\left(t\right)\right]\\ & + & iG^{*}\left[\sigma_{12}^{2}\left(t\right)-\sigma_{01}^{2}\left(t\right)\right]\;, \end{array}$$

(6e) $$\begin{array}{ccc} {\dot{\sigma }}_{12}\left(t\right) & = & \left(i\frac{E+E_{B}}{\hbar }-i\omega -\gamma_{12}\right)\sigma_{12}\left(t\right)+i\frac{\Omega^{*}\left(t\right)}{2}\left[\sigma_{22}\left(t\right)-\sigma_{11}\left(t\right)\right]+i\frac{\Omega (t)}{2}\sigma_{02}\left(t\right)\\ & + & iG^{*}\left[\sigma_{01}\left(t\right)+\sigma_{12}\left(t\right)\right]\left[\sigma_{22}\left(t\right)-\sigma_{11}\left(t\right)\right]+iG\left[\sigma_{10}\left(t\right)+\sigma_{21}\left(t\right)\right]\sigma_{02}\left(t\right)\;. \end{array}$$

## 3. Biexciton State Preparation Using on-off Pulses

In this section we derive on-off pulses which accomplish fast and efficient biexciton state preparation. In the derivation we temporarily ignore the relaxation rates and the self-interaction parameter *G*, but in the next section we test the obtained pulses by simulating the full density matrix Equation (6). We follow our recent work [43] where we obtained similar pulses but for an isolated quantum dot. Using the transformed probability amplitudes $b_{0}=c_{0},b_{1}=c_{1}e^{i\omega t},b_{2}=c_{2}e^{2i\omega t}$ for the ground, exciton and biexciton states, respectively, fixing the laser frequency at the two-photon resonance value $\hbar \omega =E+E_{B}/2$ and performing the rotating wave approximation, we find from Equations (1) and (2), with G=0 in the latter, the transformed Hamiltonian

(7) $${\tilde{H}}_{B}\left(t\right)=\hbar \left(\begin{array}{ccc} 0 & -\frac{\tilde{\Omega }\left(t\right)e^{-i\phi }}{2} & 0\\ -\frac{\tilde{\Omega }\left(t\right)e^{i\phi }}{2} & -\frac{E_{B}}{2\hbar } & -\frac{\tilde{\Omega }\left(t\right)e^{-i\phi }}{2}\\ 0 & -\frac{\tilde{\Omega }\left(t\right)e^{i\phi }}{2} & 0 \end{array}\right),$$

where the real control parameter $\tilde{\Omega }\left(t\right)$ and the constant phase ϕ are obtained from the complex Rabi frequency (3) as follows

(8) $$\Omega \left(t\right)=\tilde{\Omega }\left(t\right)e^{i\phi },\;\tilde{\Omega }\left(t\right)=\left|\Omega_{0}\right|f\left(t\right).$$

A further transformation

(9) $$a_{0}=\frac{1}{\sqrt{2}}\left(b_{2}e^{-i\phi }+b_{0}e^{i\phi }\right),\;a_{1}=b_{1},\;a_{2}=\frac{1}{\sqrt{2}}\left(b_{2}e^{-i\phi }-b_{0}e^{i\phi }\right),$$

leads to ${\dot{a}}_{2}=0$, while $a_{0},a_{1}$ satisfy the two-level system

(10) $$i\left(\begin{array}{c} {\dot{a}}_{0}\\ {\dot{a}}_{1} \end{array}\right)=\left(\begin{array}{cc} 0 & -\frac{\tilde{\Omega }\left(t\right)}{\sqrt{2}}\\ -\frac{\tilde{\Omega }\left(t\right)}{\sqrt{2}} & -\frac{E_{B}}{2\hbar } \end{array}\right)\left(\begin{array}{c} a_{0}\\ a_{1} \end{array}\right).$$

The ground state initial conditions $c_{0}\left(0\right)=1,c_{1}\left(0\right)=c_{2}\left(0\right)=0$, same for $b_{i}$, give $a_{0}\left(0\right)=e^{i\phi }/\sqrt{2},a_{1}\left(0\right)=0,a_{2}\left(0\right)=-e^{i\phi }/\sqrt{2}$. However, $a_{2}\left(t\right)=a_{2}\left(0\right)=-e^{i\phi }/\sqrt{2}$ is constant, and from Equation (9) we see that if the control $\tilde{\Omega }\left(t\right)$ is selected such that at the final time t=T it is $a_{0}\left(T\right)=-e^{i\phi }/\sqrt{2}$, then $b_{2}\left(T\right)=-e^{2i\phi }\Rightarrow \left|c_{2}\left(T\right)\right|=1$ and the biexciton state is perfectly prepared. The two-level state ${\left(a_{0}\;a_{1}\right)}^{T}$ is normalized with $1/\sqrt{2}$ instead of the usual 1, thus $a_{1}\left(T\right)=0$. It becomes obvious that the control $\tilde{\Omega }\left(t\right)$ should be chosen such that the initial and final states of the two-level system (10) differ by a π-phase, and this imposes the following condition on the propagator *U* of the system

(11) $$U=\left(\begin{array}{cc} -1 & 0\\ 0 & z \end{array}\right),$$

where *z* is indifferent.

For a constant pulse $\tilde{\Omega }\left(t\right)={\tilde{\Omega }}_{0}$ with duration *T*, propagator *U* can be easily found to be

(12) $$U=e^{i\omega_{B}T}\left(\begin{array}{cc} \cos\tilde{\omega }T-in_{z}\sin\tilde{\omega }T & -in_{x}\sin\tilde{\omega }T\\ -in_{x}\sin\tilde{\omega }T & \cos\tilde{\omega }T+in_{z}\sin\tilde{\omega }T \end{array}\right),$$

where

(13) $$\omega_{B}=\frac{E_{B}}{4\hbar }$$

and

(14) $$\tilde{\omega }=\sqrt{\omega_{B}^{2}+\frac{{\tilde{\Omega }}_{0}^{2}}{2}},\;n_{x}=-\frac{1}{\sqrt{2}}\frac{{\tilde{\Omega }}_{0}}{\tilde{\omega }},\;n_{z}=\frac{\omega_{B}}{\tilde{\omega }}.$$

Using condition (11) we can find the duration and amplitude of the fastest constant pulse which completely generates the biexciton state. Observe that for $\tilde{\omega }T=m\pi$, where *m* positive integer, the propagator (12) becomes

(15) $$U=\left(\begin{array}{cc} e^{i\omega_{B}T}\cos m\pi & 0\\ 0 & e^{i\omega_{B}T}\cos m\pi \end{array}\right).$$

From Equation (11), the upper diagonal element should satisfy $e^{i\omega_{B}T}\cos m\pi =-1$, thus $e^{i\omega_{B}T}={\left(-1\right)}^{m+1}=\pm 1$. Minimum *T* is obtained for even *m* and it is $T=\pi /\omega_{B}$. The minimum required constant amplitude ${\tilde{\Omega }}_{0}^{min}$ is obtained from $\tilde{\omega }T=m\pi$ for m=2, and we find

(16) $$T=\frac{\pi }{\omega_{B}},\;{\tilde{\Omega }}_{0}^{min}=\omega_{B}\sqrt{6}\approx 2.45\omega_{B}.$$

We next show that on-off pulse-sequences with carefully selected pulse timings can achieve perfect biexciton state prepartion in shorter times than $T=\pi /\omega_{B}$. As we previously explained, the two-level system should comes back to its starting state having obtained a π-phase. It turns out that a pulse-sequence of the form on-off-on is the simplest one to achieve this, since the first on-pulse rotates the Bloch vector away from the north pole, the intermediate off-pulse moves it parallel to the equator, and the final on-pulse rotates it back to the north pole. Let $t_{i}$, i=1,2,3 be the durations of these individual pulses. To find the minimum possible total duration $T=t_{1}+t_{2}+t_{3}$ such that the π-phase condition is satisfied, we follow Refs. [43,58] and exploit the fact that in each time interval the Hamiltonian of the two-level system is constant. The total propagator *U* can be decomposed as

(17) $$U=U_{on}^{t_{3}}U_{off}^{t_{2}}U_{on}^{t_{1}},$$

with $U_{on}^{t_{j}}$, j=1,3, obtained from Equation (12) using the corresponding timing $t_{j}$, and $U_{off}^{t_{2}}$ obtained by propagating Equation (10) with $\tilde{\Omega }\left(t\right)=0$ for duration $t_{2}$, thus

(18) $$U_{off}^{t_{2}}=e^{i\omega_{B}t_{2}}\left(\begin{array}{cc} e^{-i\omega_{B}t_{2}} & 0\\ 0 & e^{i\omega_{B}t_{2}} \end{array}\right).$$

By multiplying the 2×2 matrices we obtain the following expression for the total propagator

(19) $$U=e^{i\omega_{B}T}\left(\begin{array}{cc} v_{0}+v_{3} & v_{1}+iv_{2}\\ v_{1}-iv_{2} & v_{0}-v_{3} \end{array}\right),$$

with elements

(20a) $$\begin{array}{ccc} v_{0} & = & \cos\omega_{B}t_{2}\cos\tilde{\omega }\left(t_{1}+t_{3}\right)-n_{z}\sin\omega_{B}t_{2}\sin\tilde{\omega }\left(t_{1}+t_{3}\right), \end{array}$$

(20b) $$\begin{array}{ccc} v_{1} & = & -in_{x}\cos\omega_{B}t_{2}\sin\tilde{\omega }\left(t_{1}+t_{3}\right)+2in_{x}n_{z}\sin\tilde{\omega }t_{1}\sin\omega_{B}t_{2}\sin\tilde{\omega }t_{3}, \end{array}$$

(20c) $$\begin{array}{ccc} v_{2} & = & in_{x}\sin\omega_{B}t_{2}\sin\tilde{\omega }\left(t_{3}-t_{1}\right), \end{array}$$

(20d) $$\begin{array}{ccc} v_{3} & = & -in_{z}\cos\omega_{B}t_{2}\sin\tilde{\omega }\left(t_{1}+t_{3}\right)-i\sin\omega_{B}t_{2}\cos\tilde{\omega }\left(t_{3}-t_{1}\right)+2in_{z}^{2}\sin\tilde{\omega }t_{1}\sin\omega_{B}t_{2}\sin\tilde{\omega }t_{3}. \end{array}$$

From the requirement (11) we obtain for the off-diagonal elements $v_{1}=v_{2}=0$. The condition $v_{2}=0$ leads to the relation

(21) $$t_{3}-t_{1}=\pm \frac{\pi }{\tilde{\omega }},$$

since the other potential solutions, $t_{2}=\pi /\omega_{b}$ and $t_{1}=t_{3}$ lead to total durations $T>\pi /\omega_{B}$, as explained in Ref. [43] and can be easily verified, thus they are rejected. Using Equation (21) in the expression (20b), along with the identity sin(θ±π)=−sinθ, we find

(22) $$v_{1}=2in_{x}\sin\tilde{\omega }t_{1}\left(\cos\tilde{\omega }t_{1}\cos\omega_{B}t_{2}-n_{z}\sin\tilde{\omega }t_{1}\sin\omega_{B}t_{2}\right).$$

The requirement $v_{1}=0$ is satisfied for

(23) $$\cos\tilde{\omega }t_{1}\cos\omega_{B}t_{2}=n_{z}\sin\tilde{\omega }t_{1}\sin\omega_{B}t_{2},$$

while the other potential solution $\sin\tilde{\omega }t_{1}=0$ leads to a total durations $T>\pi /\omega_{B}$, as discussed in Ref. [43]. Using Equations (21) and (23) and the relations cos(θ±π)=−cosθ,sin(θ±π)=−sinθ, we end up with the following expression for the total propagator

(24) $$U=e^{i\omega_{B}T}\left(\begin{array}{cc} e^{i\omega_{b}\tau_{2}} & 0\\ 0 & e^{-i\omega_{b}\tau_{2}} \end{array}\right).$$

The requirement for the upper diagonal element from Equation (11) becomes $e^{i\omega_{b}\left(T+\tau_{2}\right)}=-1=e^{i\pi }$, consequently

(25) $$T+t_{2}=t_{1}+2t_{2}+t_{3}=\frac{\pi }{\omega_{B}},$$

leading to a total duration satisfying $T<\pi /\omega_{b}$, thus shorter than the minimum necessary duration of a constant pulse for complete biexciton state preparation. Now observe that Equations (21), (23) and (25) comprise a system with unknowns the timings $t_{i},i=1,2,3$ of the individual pulses. If we exploit Equations (21) and (25) to express $t_{1},t_{3}$ in terms of $t_{2}$, we obtain

(26) $$t_{1}=\frac{\pi }{2}\left(\frac{1}{\omega_{B}}\mp \frac{1}{\tilde{\omega }}\right)-t_{2},\;t_{3}=\frac{\pi }{2}\left(\frac{1}{\omega_{B}}\pm \frac{1}{\tilde{\omega }}\right)-t_{2}.$$

Furthermore, if we substitute in Equation (23) the expression for $t_{1}$ from Equation (26) and additionally use the identity tan(θ±π/2)=−cotθ, then we find that the duration $t_{2}$ of the middle off-pulse satisfies the transcendental equation

(27) $$\tan\left[\tilde{\omega }\left(\frac{\pi }{2\omega_{B}}-t_{2}\right)\right]=-n_{z}\tan\omega_{B}t_{2}.$$

The expressions with the ± signs in Equation (26) indicate that the durations of the initial and final on-pulses can be interchanged. For the transcendental Equation (27) to have at least one solution, the pulse-sequence amplitude should be larger than the threshold value ${\tilde{\Omega }}_{0}^{min}=\omega_{B}\sqrt{6}$, which is found by setting $t_{2}=0$. We focus our attention in the range ${\tilde{\Omega }}_{0}>{\tilde{\Omega }}_{0}^{min}$, since such values can be easily obtained in experiments and also lead to durations $T<\pi /\omega_{B}$, the shorter duration achieved with a constant pulse. Please note that for larger ${\tilde{\Omega }}_{0}$, Equation (27) can have more solutions, in which case we pick the larger $t_{2}$, corresponding to the shorter total duration $T=\pi /\omega_{B}-t_{2}$. For very large values of ${\tilde{\Omega }}_{0}$, the shortest duration tends to the limiting value $\pi /(2\omega_{B})$. In Ref. [43] we have shown using numerical optimal control that when the control input is bounded as $0\leq \tilde{\Omega }\left(t\right)\leq {\tilde{\Omega }}_{0}$ and for maximum control amplitude spanning a wide range of values, $\sqrt{6}\omega_{B}\leq {\tilde{\Omega }}_{0}\leq 50\omega_{B}$, the three-segment pulse-sequence derived here prepares the biexciton state in minimum time.

## 4. Numerical Results for the Coupled SQD-MNP System

**Figure 2:**
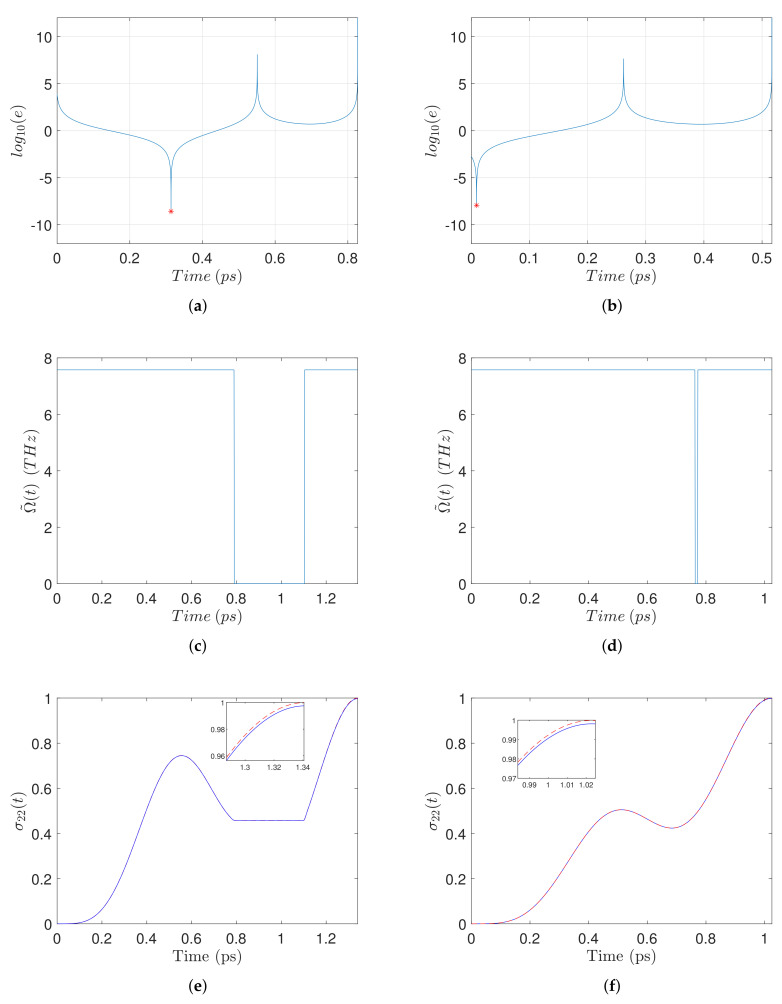
Left-column top to bottom: (**a**) Logarithm of the squared error between the two sides of transcendental Equation (27) as a function of the duration $t_{2}$ of the intermediate (off) pulse. The negative resonance indicated with red star corresponds to the solution. (**c**) Corresponding on-off pulse-sequence. (**e**) Time evolution of biexciton population $\sigma_{22}\left(t\right)$ (blue solid line) obtained by simulating the full Equation (6) with input the above pulse-sequence. For comparison, the red dashed line shows $\sigma_{22}\left(t\right)$ if relaxation is ignored from the system equations. These results are obtained for interparticle distance R=14 nm and biexciton energy shift $E_{B}=5$ meV. In the right-column (**b**,**d**,**f**) we display similar plots for the case with the same R=14 nm but larger $E_{B}=8$ meV.

To solve the transcendental Equation (27), we plot the logarithm of the squared error

(28) $$e={\left|\tan\left[\tilde{\omega }\left(\frac{\pi }{2\omega_{B}}-t_{2}\right)\right]+n_{z}\tan\omega_{B}t_{2}\right|}^{2}$$

as a function of $t_{2}$. The negative resonances of this plot correspond to the solutions of the transcendental equation. For example, in Figure 2a we plot this logarithmic error for the case where R=14 nm and $E_{B}=5$ meV. There is only one negative resonance, marked with a red star, giving the duration $t_{2}$ of the intermediate off-pulse. The corresponding on-off-on pulse-sequence is displayed in Figure 2c. We use this pulse-sequence to simulate the full set of the density matrix Equation (Section 2). For the relaxation rates encountered in these equations we use the values $\Gamma_{11}^{-1}=\Gamma_{22}^{-1}=800$ ps and $\gamma_{01}^{-1}=\gamma_{02}^{-1}=\gamma_{12}^{-1}=300$ ps. For the other parameters which are necessary in the simulation, we use the values $\varepsilon_{env}=\varepsilon_{0}$, $\varepsilon_{S}=6\varepsilon_{0}$, $r_{mnp}=7.5$ nm, μ=0.65 enm, $\hbar \omega_{0}=2.5$ eV, and $\varepsilon_{S}=6\varepsilon_{0}$, which are typical for colloidal quantum dots, such as the CdSe-based SQD. For the biexciton binding energy $E_{B}$ we use values in the range of a few meV, which are typical for semiconductor quantum dots with the optical gap considered here, see for example Ref. [59]. The same values also appear in many other works [4,5,6,7,12,17,18,20,21,60]. For the nanoparticle dielectric constant $\varepsilon_{m}\left(\omega \right)$, we make use of the experimental data which are available for gold [61]. The value of the applied external field is such that $\mu E_{0}/\hbar =15$ THz. We additionally emphasize that a nonzero self-interaction parameter *G* is used in the simulations, obtained from Equation (4). In Figure 2e we display the time evolution of the biexciton population $\sigma_{22}\left(t\right)$ (blue solid line), for the pulse-sequence shown in Figure 2c, corresponding to the case R=14 nm and $E_{B}=5$ meV. From the detail shown in the inset, we observe that a fidelity higher than 0.99 is achieved, even when relaxation and self-interaction are taken into account. In order to identify the contribution of each mechanism to the deviation from perfect population transfer, we also plot $\sigma_{22}\left(t\right)$ for the case where the relaxation rates are set to zero but G≠0 (red dashed line). Comparing the two plots we conclude that the major efficiency limitation in the specific example is due to relaxation. In the second column of Figure 2 we display the solution of the transcendental equation, the on-off pulse sequence, and the time evolution of the biexciton population, for the same interparticle distance R=14 nm and a larger biexciton energy $E_{B}=8$ meV. The value of *R* determines the maximum control amplitude ${\tilde{\Omega }}_{0}$ in the pulse sequence, which is thus the same for both examples, see Figure 2c,d. From the same figures we observe that for the larger value of $E_{B}$ the duration of the intermediate off pulse becomes shorter and the pulse-sequence is approaching the constant pulse shape. The total duration is also smaller and thus the detrimental effect of relaxation and nonlinearity *G* (which has the same value for both cases since *R* is the same) is reduced, leading to a higher final biexciton population, as revealed by carefully comparing the insets of Figure 2e,f.

**Figure 3:**
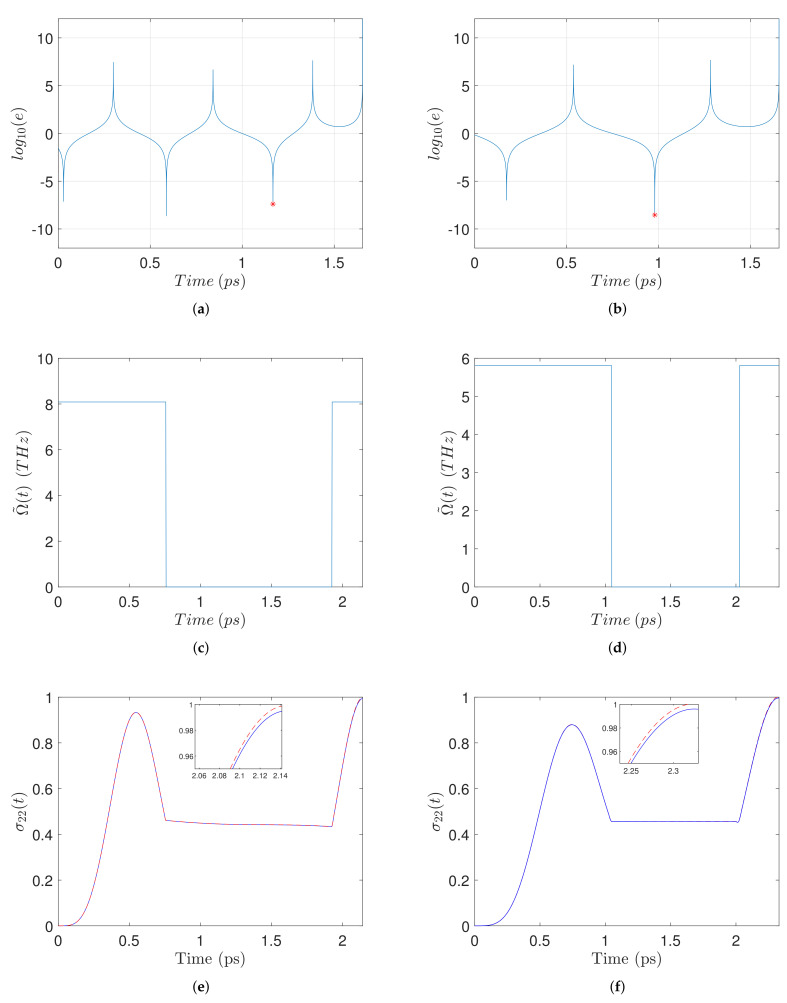
Left-column top to bottom: (**a**) Logarithmic squared error (28), (**c**) pulse-sequence, and (**e**) time evolution of $\sigma_{22}\left(t\right)$, for R=13 nm and $E_{B}=2.5$ meV. In the right-column (**b**,**d**,**f**) we display similar plots for the case with a larger interparticle distance R=30 nm but the same biexciton energy shift $E_{B}=2.5$ meV.

**Figure 4:**
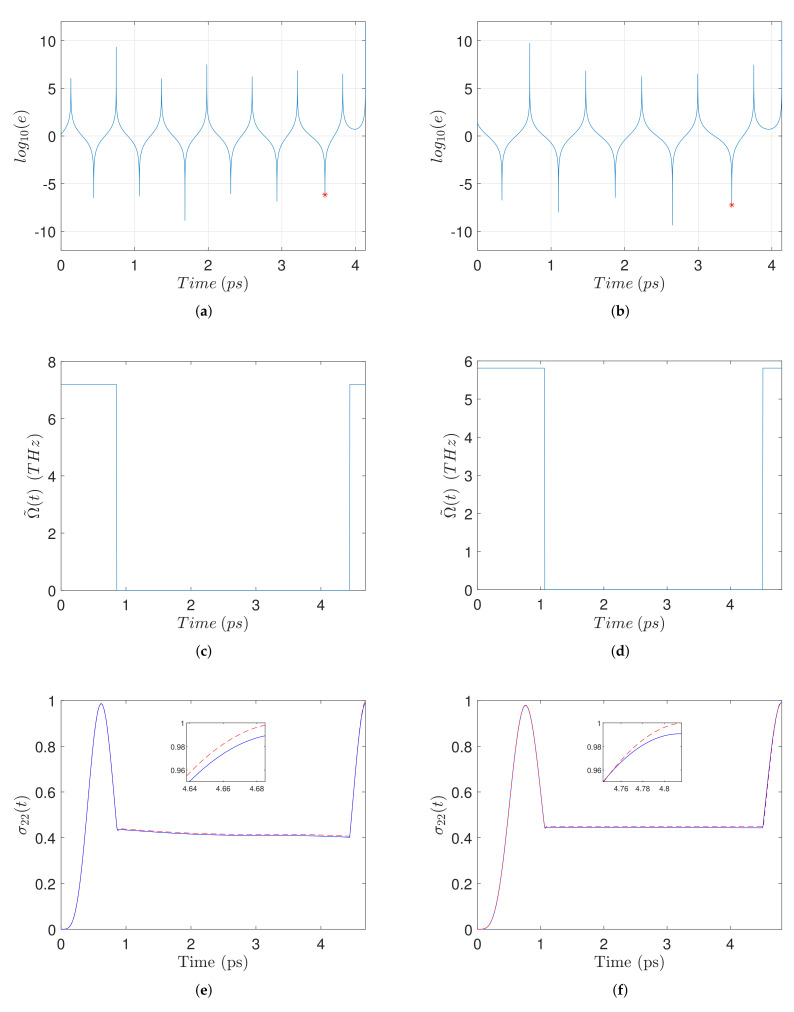
Left-column top to bottom: (**a**) Logarithmic squared error (28), (**c**) pulse-sequence, and (**e**) time evolution of $\sigma_{22}\left(t\right)$, for R=15 nm and $E_{B}=1$ meV. In the right-column (**b**,**d**,**f**) we display similar plots for the case with a larger interparticle distance R=30 nm but the same biexciton energy shift $E_{B}=1$ meV.

In Figure 3 we present similar plots for two different values of the interparticle distance, R=13 nm (left column) and R=30 nm (right column), and a common biexciton energy $E_{B}=2.5$ meV. Observe from Figure 3a,b that for these examples the transcendental equation has more solutions (three and two, respectively, corresponding to the negative resonances). For each case we pick as $t_{2}$ the largest solution, indicated by a red star, leading to the smallest total duration $T=\pi /\omega_{B}-t_{2}$, see Equation (25). Regarding now the resultant pulse-sequences, we observe from Figure 3c,d that the case with smaller *R* has a larger pulse amplitude, see Equation (3), and a shorter duration. Both durations are longer than those of the pulse-sequences in Figure 2, since the biexciton energy used here is smaller. Although for the case with smaller interparticle distance the pulse amplitude is larger and the pulse duration is shorter, the biexciton population generated at the final time is slightly smaller, as can be observed from the details in Figure 3e,f. The reason is that for smaller *R* the self-interaction parameter *G* is larger, see Equation (4), and this compensates for the previously mentioned characteristics of the pulse-sequence which otherwise would lead to a better performance. The negative effect of smaller *R* is also revealed by comparing the red dashed lines in the two insets, since recall that they are obtained using zero relaxation rates. Similar conclusions can be drawn from Figure 4, where we display analogous results for the cases with R=15 nm (left column) and R=30 nm (right column), with a common biexciton energy $E_{B}=1$ meV, smaller than in the previous cases. The interesting thing to observe here is that the transcendental equation has even more solutions (Figure 4a,b), while the pulse-sequence durations are longer (Figure 4c,d), leading to worse performance than in the previous cases (Figure 4e,f). We point out that the negative resonances in Figure 2a,b, Figure 3a,b and Figure 4a,b correspond to solutions of the transcendental Equation (27), while the variability of the negative peaks is just due to numerical errors.

**Figure 5:**
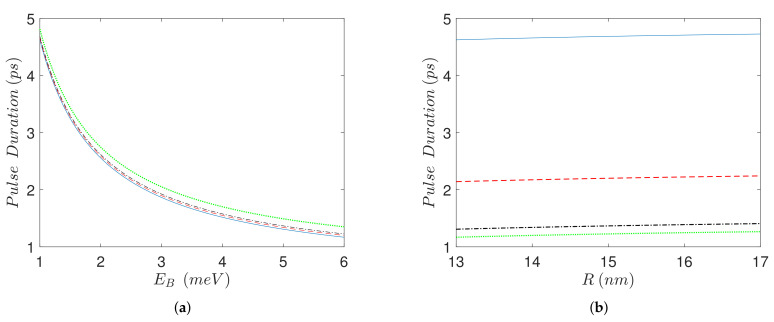
(**a**) Pulse-sequence duration as a function of biexciton energy shift $E_{B}$, for four values of the interparticle distance, R=13 nm (blue solid line), R=14 nm (red dashed line), R=15 nm (black dashed-dotted line), R=30 nm (green dotted line). (**b**) Pulse-sequence duration as a function of interparticle distance *R*, for four values of the biexciton energy shift, $E_{B}=1$ meV (blue solid line), $E_{B}=2.5$ meV (red dashed line), $E_{B}=5$ meV (black dashed-dotted line), $E_{B}=6$ meV (green dotted line).

In all the presented examples we see that, for smaller values of the biexciton energy shift $E_{B}$, the pulse-sequences needed for biexciton state preparation are longer. This is also illustrated in Figure 5a, where the pulse-sequence duration is plotted versus the biexciton energy shift, for various values of the SQD-MNP distance *R*. The reason is that $E_{B}$ determines the energy separation between the exciton and biexciton states, and thus to discriminate between them for smaller $E_{B}$ a longer duration is required. The biexciton energy shift essentially determines the quantum speed limit of the system under the considered control (the amplitude of the applied field). In Figure 5b we plot the pulse-sequence duration as a function of the interparticle distance *R*, for various values of the biexciton energy shift. Please observe that, in all the cases, the duration slightly increases with *R*. The reason behind this increase is that for larger *R* the pulse amplitude decreases, see Equation (3), thus a longer pulse-sequence is necessary in order to accomplish the desired transfer.

**Figure 6:**
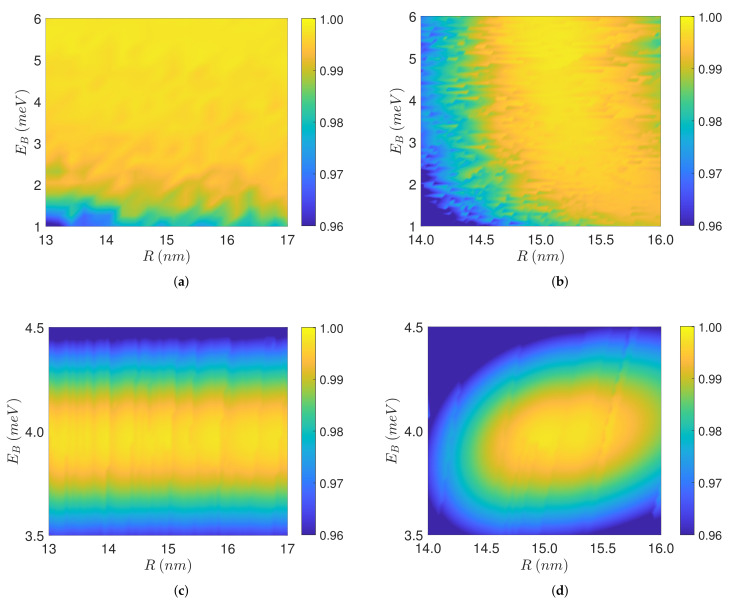
(**a**) Final value of the population $\sigma_{22}\left(T\right)$ from the numerical solution of Equation (6) using the pulse-sequence obtained for each pair of values $\left(E_{B},R\right)$ in the range shown. (**b**) $\sigma_{22}\left(T\right)$ for the pulse timings obtained with the fixed reference value $R_{0}=15$ nm and variable $E_{B}$, when applied to system Equation (6) with variable *R* in the range $R_{0}\pm 1$ nm and the corresponding $E_{B}$. (**c**) $\sigma_{22}\left(T\right)$ for the pulse timings obtained with the fixed reference value $E_{B}^{0}=4$ meV and variable *R*, when applied to Equation (6) with variable $E_{B}$ in the range $E_{B}^{0}\pm 0.5$ meV and the corresponding *R*. (**d**) $\sigma_{22}\left(T\right)$ for the pulse-sequence obtained with fixed $R_{0}=15$ nm and $E_{B}^{0}=4$ meV, when applied to Equation (6) with variable *R* in the range $R_{0}\pm 1$ nm and $E_{B}$ in the range $E_{B}^{0}\pm 0.5$ meV.

In Figure 6a we display the final biexciton population $\sigma_{22}\left(T\right)$ achieved for values of the interparticle distance *R* and biexciton energy shift $E_{B}$ in the intervals shown. For each pair $\left(R,E_{B}\right)$ we find the corresponding pulse-sequence and apply it to the coupled SQD-MNP system Equation (6). Observe that high-levels of fidelity are obtained for a wide range of these parameter values. The efficiency generally increases with increasing *R* since the self-interaction parameter *G* decreases, see Equation (4), something that was also observed in the examples shown in Figure 3 and Figure 4. Nevertheless, there are some fluctuations in the efficiency as *R* increases, which can be attributed to the reduction of the effective pulse amplitude, Equation (3), and the corresponding slight increase of the pulse-sequence duration, displayed in Figure 5b. With respect to growing $E_{B}$, the efficiency also generally increases since the pulse-sequence duration is reduced, see Figure 5a and the example of Figure 2. In this case, the efficiency fluctuations can be explained since, for larger $E_{B}$, the resultant pulse-sequences deviate from the more robust composite pulse form, Figure 2c, and approach the constant pulse shape, Figure 2d, which is more vulnerable to perturbations such as the *R*-dependent *G*-term. For this reason the fluctuations with increasing $E_{B}$ are more intense for smaller *R*.

To evaluate the robustness of the proposed method against a positioning error of the MNP, we find the pulse timings for a fixed reference value $R_{0}=15$ nm of the interparticle distance and variable $E_{B}$, and apply the resultant pulse-sequence in system Equation (6) with variable *R* in the range $R_{0}\pm 1$ nm and the corresponding $E_{B}$. The final biexciton population obtained in this case is displayed in Figure 6b, where observe that despite the imperfections high levels of fidelity can be still achieved, especially for larger interparticle distances and biexciton energy shifts. We investigate next the robustness with respect to variations in $E_{B}$. Analogously with the previous case, we find the pulse timings for fixed $E_{B}^{0}=4$ meV and variable *R*, and then find numerically the final biexciton population for $E_{B}$ in the range $E_{B}^{0}\pm 0.5$ meV and the corresponding *R*. The results are shown in Figure 6c, where observe that large efficiency is obtained in an appreciable belt around the central value $E_{B}^{0}$. The robustness belt extends more in the area $E_{B}<E_{B}^{0}$, since the pulse-sequences used are not tuned with respect to the biexciton energy shift, as they were in the previous figures, thus larger values of $E_{B}$ cause larger deviations from perfect transfer. Finally, in Figure 6d we consider a combined error in both *R* and $E_{B}$, with the pulse timings obtained for fixed $R_{0}=15$ nm and $E_{B}^{0}=4$ meV. Observe that, despite the simultaneous presence of the errors, a noticeable robustness area still survives in parameter space.

## 5. Conclusions

We studied the problem of pulsed biexciton state preparation in a SQD-MNP system and showed with numerical simulations that, when using a on-off-on pulse-sequence with carefully selected pulse durations, the desired state can be efficiently prepared for a broad range of SQD-MNP (interparticle) distances and various values of the biexciton energy shift. We find that the transfer fidelity is in general better for larger values of the interparticle distance, because then the influence of the nanoparticle is weaker, and larger values of the biexciton energy shift, since the energy separation of the exciton and biexciton levels is then larger. The obtained fidelities are robust against small variations in the values of these parameters. Our results can find applications in the emerging area of nanomaterials and nanosystems for quantum technologies.

Closing, we point out that the presence of the MNP can also modify the effective damping of SQD, giving rise to further peculiar effects, see for example [62]. Since the applied pulses have been obtained independently of the relaxation mechanisms affecting SQD, an interesting future work would be to investigate their performance when taking these phenomena into account. Another possible test is to evaluate the efficiency of obtained pulses using different relaxation rates for the excited states and different dipole moments for the ground to exciton and exciton to biexciton transitions, as in the model of [54]. Of course, with the accumulation of all these imperfections, it is probably better to recourse to numerical optimization in order to find the pulses which optimize the biexciton transfer, as we recently did for the fast spin initialization of a quantum dot coupled to a two-dimensional nanostructure [63,64,65].

## Data Availability

Data is contained within the article.

## Abbreviations

The following abbreviations are used in this manuscript:

SQD	Semiconductor Quantum Dot
MNP	Metal Nanoparticle
